# Large-scale application of free energy perturbation calculations for antibody design

**DOI:** 10.1038/s41598-022-14443-z

**Published:** 2022-07-21

**Authors:** Fangqiang Zhu, Feliza A. Bourguet, William F. D. Bennett, Edmond Y. Lau, Kathryn T. Arrildt, Brent W. Segelke, Adam T. Zemla, Thomas A. Desautels, Daniel M. Faissol

**Affiliations:** 1 Biosciences and Biotechnology Division, Physical and Life Sciences Directorate, Lawrence Livermore National Laboratory, Livermore, USA; 2 Global Security Computing Division, Computing Directorate, Lawrence Livermore National Laboratory, Livermore, USA; 3 Computational Engineering Division, Engineering Directorate, Lawrence Livermore National Laboratory, Livermore, USA

**Keywords:** Computational chemistry, Molecular dynamics

## Abstract

Alchemical free energy perturbation (FEP) is a rigorous and powerful technique to calculate the free energy difference between distinct chemical systems. Here we report our implementation of automated large-scale FEP calculations, using the Amber software package, to facilitate antibody design and evaluation. In combination with Hamiltonian replica exchange, our FEP simulations aim to predict the effect of mutations on both the binding affinity and the structural stability. Importantly, we incorporate multiple strategies to faithfully estimate the statistical uncertainties in the FEP results. As a case study, we apply our protocols to systematically evaluate variants of the m396 antibody for their conformational stability and their binding affinity to the spike proteins of SARS-CoV-1 and SARS-CoV-2. By properly adjusting relevant parameters, the particle collapse problems in the FEP simulations are avoided. Furthermore, large statistical errors in a small fraction of the FEP calculations are effectively reduced by extending the sampling, such that acceptable statistical uncertainties are achieved for the vast majority of the cases with a modest total computational cost. Finally, our predicted conformational stability for the m396 variants is qualitatively consistent with the experimentally measured melting temperatures. Our work thus demonstrates the applicability of FEP in computational antibody design.

## Introduction

Alchemical free energy perturbation (FEP) simulation^1^ is a rigorous physics-based method to calculate the free energy difference between distinct chemical systems. Due to recent technological advancement, FEP is now capable of accurately predicting relative binding affinities^2–4^ and has found increasingly more applications in drug development.

Antibodies are proteins produced by the immune system to bind specific antigenic proteins and thereby neutralize the antigen. Therapeutically, antibodies can be designed and manufactured as effective medicines against the infection. Recently, FEP has been successfully applied to predict the relative binding affinity between antibody and antigen^5,6^, thus potentially facilitating antibody design^7–9^ and optimization.

In our ongoing battle against the current COVID19 pandemic^10^, we aim to develop antibodies to neutralize SARS-CoV-2 and other coronaviruses. Toward this goal, we introduced FEP simulations as one of the computational tools for our multidisciplinary team. Specifically, the basic task of FEP is to predict the change in the binding affinity due to proposed mutations on the antibody. In our workflow, batches of mutations are routinely proposed for evaluation, and quick turnarounds are necessary for further decision making. Such requirements necessitate automated processing of many FEP calculations. In this situation, it is not feasible to manually examine all the simulations individually and identify potential problems therein. Therefore, a faithful and automated estimation of the uncertainties in such calculations is especially important, as it could provide the level of confidence for the FEP results when data from many different sources are integrated to inform the decision making. With these requirements in mind, we implemented automated protocols for performing FEP calculations with Hamiltonian replica exchange^11^ using the Amber software package^12^, along with an uncertainty estimation that incorporates a number of factors in the analysis of the simulations.

Whereas the binding affinity to antigen is a critical component for the efficacy of an antibody, the structural stability is important for the developability^13^, such as manufacture, storage, and distribution of the antibody. Therefore, computational stability evaluation^14^ is desired in our antibody design. Recent studies demonstrated that FEP^15–18^ and other computational approaches^19–24^ could provide reasonable predictions for the relative stability of protein mutants. In this project, we also incorporated stability prediction by FEP calculations, such that our FEP protocols evaluate the effect of proposed mutation on both the binding affinity and the conformational stability of the antibody.

In this report, we take the m396 antibody^25^ as a case study to demonstrate our FEP protocols. m396 is known to neutralize the coronavirus SARS-CoV-1 by binding to the receptor binding domain (RBD) of the viral spike protein^25^, but does not bind or neutralize SARS-CoV-2^26^. The RBD is a self-contained and stable domain, and isolated RBDs could independently bind m396 without other parts of the spike protein^25^. One of our objectives is to modify relevant residues of m396 such that the mutated antibody could bind the SARS-CoV-2 RBD. The focus of this article is only on the implementation of FEP calculations, and our much broader efforts in the antibody design will be reported in due course.

## Methods

**Figure 1:**
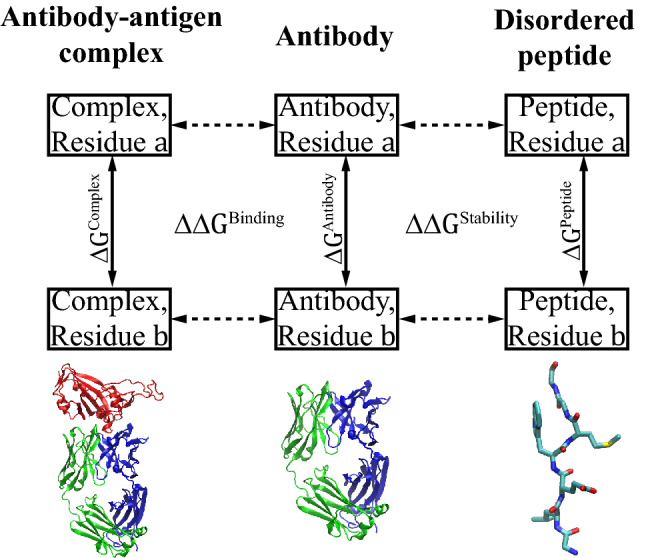
Schematics for the FEP calculations in this study. The free energy differences ${\Delta G}^{\mathrm{Complex}}$, ${\Delta G}^{\mathrm{Antibody}}$, and ${\Delta G}^{\mathrm{Peptide}}$ between systems with residues $a$ and $b$ are obtained from the corresponding FEP simulations, and the results further determine (Eqs. (1) and (2)) the effect of mutation on the binding (${\Delta \Delta G}^{\mathrm{Binding}}$) and the stability (${\Delta \Delta G}^{\mathrm{Stability}}$) of the antibody. The illustrated protein structures are from the m396 antibody^25^, rendered by the VMD program^43^ (version 1.9.3).

In this study, the basic task of an FEP calculation is to evaluate two effects due to the mutation of a single residue on the antibody from type $a$ to type $b$, as schematized in Fig. 1. The first effect, quantified by

1 $${\Delta \Delta G}^{\mathrm{Binding}}= {\Delta G}^{\mathrm{Complex}}-{\Delta G}^{\mathrm{Antibody}},$$

describes the shift in equilibrium between the bound and unbound states. Here ${\Delta G}^{\mathrm{Complex}}$ is the difference in free energy between the bound systems with the concerned residue in type $a$ and type $b$, respectively. Similarly, ${\Delta G}^{\mathrm{Antibody}}$ is the free energy difference between the unbound antibody systems of $a$ and $b$. The difference between the two $\Delta G$ s above, ${\Delta \Delta G}^{\mathrm{Binding}}$, directly determines the change in the binding affinity due to the mutation. The second effect, quantified by

2 $${\Delta \Delta G}^{\mathrm{Stability}}={\Delta G}^{\mathrm{Antibody}}-{\Delta G}^{\mathrm{Peptide}},$$

describes the shift in equilibrium between the native (folded) and denatured (unfolded) conformations of the antibody. The structure of denatured antibody is unknown and is thus approximated by a highly simplified model of 7-residue peptide^18^ here.

Because our FEP simulations are under constant volume, the results here correspond to the differences in the Helmholtz free energy ($A$). In contrast, the thermodynamics of constant-pressure systems should be described by the Gibbs free energy ($G$), with $G=A+PV$, where $P$ and $V$ are respectively the pressure and the volume. However, given that the contribution of the $PV$ term to the $\Delta \Delta G$ is typically small for biochemical systems, we consider $\Delta \Delta A\approx \Delta \Delta G$ and use $A$ and $G$ interchangeably in this article.

In this section, we will first review the fundamental theories involved in FEP calculations. We will then describe our specific FEP simulations to evaluate the m396 antibody variants for their conformational stability and their binding to the SARS-CoV-1 and SARS-CoV-2 RBDs. We will also describe our experiments to measure the thermal stability of the antibodies.

### Free energy difference between two systems

Consider two systems $i$ and $j$, both consisting of an identical set of atoms but with different energy functions ${E}_{i}\left(\overrightarrow{X}\right)$ and ${E}_{j}\left(\overrightarrow{X}\right)$, respectively, where $\overrightarrow{X}$ represents the microstate of the system, i.e., the positions and velocities of all the atoms. Under constant temperature ($T$) and volume, the difference in the Helmholtz free energy between the two systems is given by:

3 $${A}_{j}-{A}_{i}=-{k}_{B}T\mathrm{ln}\frac{\int \mathrm{exp}\left[-{E}_{j}\left(\overrightarrow{X}\right)/{k}_{B}T\right]d\overrightarrow{X}}{\int \mathrm{exp}\left[-{E}_{i}\left(\overrightarrow{X}\right)/{k}_{B}T\right]d\overrightarrow{X}},$$

where ${k}_{B}$ is the Boltzmann constant.

The free energy difference above can be rewritten in a more practical and useful form:

4 $${A}_{j}-{A}_{i}=-{k}_{B}T\mathrm{ln}{\left\langle \mathrm{exp}\left[-\frac{{E}_{j}\left(\overrightarrow{X}\right)-{E}_{i}\left(\overrightarrow{X}\right)}{{k}_{B}T}\right]\right\rangle }_{i},$$

in which ${\langle \rangle }_{i}$ represents the expected value in the equilibrium ensemble of system $i$. This expected value can be estimated as the average of $\mathrm{exp}\left(-\frac{{\Delta E}_{ij}}{{k}_{B}T}\right)$, where ${\Delta E}_{ij}\left(\overrightarrow{X}\right)\equiv {E}_{j}\left(\overrightarrow{X}\right)-{E}_{i}\left(\overrightarrow{X}\right)$, with $\overrightarrow{X}$ taken from an equilibrium sampling of system $i$ using, e.g., molecular dynamics (MD) simulations. This method^27^ is termed exponential averaging (EXP). Alternatively, if one performs equilibrium sampling for system $j$ instead, the free energy can also be obtained in a similar way:

5 $${A}_{j}-{A}_{i}={k}_{B}T\mathrm{ln}{\left \langle \mathrm{exp}\left[-\frac{{E}_{i}\left(\overrightarrow{X}\right)-{E}_{j}\left(\overrightarrow{X}\right)}{{k}_{B}T}\right] \right \rangle }_{j},$$

with the expected value above estimated as the average of $\mathrm{exp}\left(-\frac{{\Delta E}_{ji}}{{k}_{B}T}\right)$, where ${\Delta E}_{ji}={E}_{i}\left(\overrightarrow{X}\right)-{E}_{j}\left(\overrightarrow{X}\right)=-{\Delta E}_{ij}$.

If equilibrium sampling is available for both systems $i$ and $j$, the Bennett acceptance ratio (BAR) method^28^ can also provide the free energy difference $\Delta A\equiv {A}_{j}-{A}_{i}$, with better statistical accuracy. The BAR method is based on the following relation:

6 $${\left \langle \frac{1}{1+\mathrm{exp}\left[\frac{{\Delta E}_{ij}\left(\overrightarrow{X}\right)-\Delta A}{{k}_{B}T}\right]} \right \rangle }_{i}={\left\langle \frac{1}{1+\mathrm{exp}\left[\frac{{\Delta E}_{ji}\left(\overrightarrow{X}\right)+\Delta A}{{k}_{B}T}\right]}\right \rangle }_{j}.$$

Again, the expected values above can be estimated as the corresponding averages, with ${\Delta E}_{ij}\left(\overrightarrow{X}\right)$ and ${\Delta E}_{ji}\left(\overrightarrow{X}\right)$ obtained from the sampled microstates in systems $i$ and $j$, respectively. Equation (6) thus contains only one unknown, $\Delta A$, which can be numerically solved. If the sample sizes for systems $i$ and $j$ are different, Eq. (6) could be slightly modified into a more general form that minimizes the statistical error^28^. In this study, since the two involved systems always have identical sample size, Eq. (6) is the optimal form to calculate the $\Delta A$.

### Sampling with intermediate windows

Although the free energy methods in “Free energy difference between two systems” are correct and rigorous, they are only practically useful if the two systems are sufficiently similar such that their equilibrium distributions of the microstates $\overrightarrow{X}$ are largely overlapped. If the two systems are substantially different, direct applications of Eqs. (4)–(6) will be practically difficult to achieve reliable results. In such cases, one could introduce intermediate systems (each conventionally called a “window”), calculate the free energy difference between adjacent systems using the methods in “Free energy difference between two systems”, and then add these increments to obtain the free energy difference between the two end systems.

Suppose we aim to calculate the free energy difference between two dissimilar systems, $a$ and $b$, with the same set of atoms. Typically, intermediate systems can be constructed by introducing a parameter $\lambda$, in the range of $\left[\mathrm{0,1}\right]$, into the energy function $E\left(\overrightarrow{X};\lambda \right)$, such that $E\left(\overrightarrow{X};\lambda =0\right)={E}_{a}\left(\overrightarrow{X}\right)$ and $E\left(\overrightarrow{X};\lambda =1\right)={E}_{b}\left(\overrightarrow{X}\right)$. Now the system free energy $A\left(\lambda \right)$ is also a function of $\lambda$, and the target free energy difference becomes ${A}_{b}-{A}_{a}=A\left(\lambda =1\right)-A\left(\lambda =0\right)$. Intermediate systems between $a$ and $b$ can thus be defined by a series of $\lambda$ values: $0={\lambda }_{1}<{\lambda }_{2}<\dots <{\lambda }_{N}=1$, such that systems $i$ and $i+1$ are sufficiently similar. With all the incremental changes $A\left({\lambda }_{i+1}\right)-A\left({\lambda }_{i}\right)$ calculated, their sum then corresponds to ${A}_{b}-{A}_{a}$.

As another alternative to calculate the free energy difference, thermodynamic integration (TI) is based on the following relation:

7 $$\frac{d}{d\lambda }A\left(\lambda \right)={\bigg\langle \frac{\partial E\left(\overrightarrow{X};\lambda \right)}{\partial \lambda }\bigg\rangle }_{\lambda },$$

in which ${\langle \rangle }_{\lambda }$ represents the expected value in the system with the energy function $E\left(\overrightarrow{X};\lambda \right)$. Therefore,

8 $${A}_{b}-{A}_{a}=\underset{0}{\overset{1}{\int }}{\langle \frac{\partial E\left(\overrightarrow{X};\lambda \right)}{\partial \lambda }\rangle }_{\lambda }d\lambda .$$

The simulation setup for TI is identical to that for FEP, both entailing equilibrium sampling in systems $E\left(\overrightarrow{X};{\lambda }_{i}\right)$ for $i$=1,2,…,$N$. To apply TI, we estimate ${f}_{i}\equiv {\langle \frac{\partial E\left(\overrightarrow{X};\lambda \right)}{\partial \lambda }\rangle }_{{\lambda }_{i}}$ by averaging the $\frac{\partial E\left(\overrightarrow{X};\lambda \right)}{\partial \lambda }$ value over the sampled microstates for each system $i$. Then a numerical integration of Eq. (8) using the trapezoidal rule will provide the free energy difference:

9 $${A}_{b}-{A}_{a}\approx \sum_{i=1}^{N-1}\frac{1}{2}\left({f}_{i}+{f}_{i+1}\right)\left({\lambda }_{i+1}-{\lambda }_{i}\right).$$

### Energy function in alchemical system

To obtain the free energy difference between two systems ($a$ and $b$) that do not share an identical atom set, an “alchemical” system can be constructed. All the atoms in $a$ and $b$ are present in the alchemical system. In Amber^12^, the microstate of an alchemical system is described as $\overrightarrow{X}=\left({\overrightarrow{X}}_{0},{\overrightarrow{X}}_{a}^{\mathrm{SC}},{\overrightarrow{X}}_{b}^{\mathrm{SC}}\right)$. Here ${\overrightarrow{X}}_{0}$ represents the common atoms in both systems. In addition, two separate sets of coordinates, ${\overrightarrow{X}}_{a}^{\mathrm{SC}}$ and ${\overrightarrow{X}}_{b}^{\mathrm{SC}}$, represent the atoms unique to systems $a$ and $b$, respectively, which are called “soft core” (SC) atoms.

The system energy is the sum of kinetic energy and potential energy. Kinetic energy is independent of $\lambda$ and thus does not contribute to the energy difference $\Delta E$. Therefore, we only need to consider the potential energy $V$ in the calculations. In Amber^12^, the potential energy for the alchemical system has the following form:

10 $$V\left(\overrightarrow{X};\lambda \right)=\left(1-\lambda \right)\cdot {V}_{a}\left(\overrightarrow{X};\lambda \right)+\lambda \cdot {V}_{b}\left(\overrightarrow{X};\lambda \right).$$

In the potential ${V}_{a}$ above, the atoms in ${\overrightarrow{X}}_{b}^{\mathrm{SC}}$ have no nonbond interaction with ${\overrightarrow{X}}_{0}$ or ${\overrightarrow{X}}_{a}^{\mathrm{SC}}$ and are thus decoupled from the rest of the system. Similarly, in ${V}_{b}$, the ${\overrightarrow{X}}_{a}^{\mathrm{SC}}$ atoms are decoupled from the rest of the system. Furthermore, the dependence of ${V}_{a}$ on $\left({\overrightarrow{X}}_{0},{\overrightarrow{X}}_{a}^{\mathrm{SC}}\right)$ and the dependence of ${V}_{b}$ on $\left({\overrightarrow{X}}_{0},{\overrightarrow{X}}_{b}^{\mathrm{SC}}\right)$ conform to the standard potentials in the MD force field for systems $a$ and $b$, respectively, except that the nonbond interactions involving the SC atoms are modified as described below, to avoid the “end-point catastrophes”.

Specifically, between an atom in ${\overrightarrow{X}}_{0}$ and another atom in ${\overrightarrow{X}}_{a}^{\mathrm{SC}}$, the van der Waals (VDW) term in ${V}_{a}$ has the modified form:

11a $${u}_{a}^{\mathrm{VDW}}\left({r}_{ij};\lambda \right)=4\varepsilon \left\{\frac{1}{{\left[\alpha \lambda +{\left({r}_{ij}/\sigma \right)}^{6}\right]}^{2}}-\frac{1}{\alpha \lambda +{\left({r}_{ij}/\sigma \right)}^{6}}\right\},$$

where ${r}_{ij}$ is the distance between the two atoms and $\alpha$ is a user-defined parameter. When $\alpha =0$ or $\lambda =0$, ${u}_{a}^{\mathrm{VDW}}$ recovers the standard Lennard–Jones potential with the parameters $\sigma$ and $\varepsilon$. Similarly, between an atom in ${\overrightarrow{X}}_{0}$ and another atom in ${\overrightarrow{X}}_{b}^{\mathrm{SC}}$, the VDW term in ${V}_{b}$ is:

11b $${u}_{b}^{\mathrm{VDW}}\left({r}_{ij};\lambda \right)=4\varepsilon \left\{\frac{1}{{\left[\alpha \left(1-\lambda \right)+{\left({r}_{ij}/\sigma \right)}^{6}\right]}^{2}}-\frac{1}{\alpha \left(1-\lambda \right)+{\left({r}_{ij}/\sigma \right)}^{6}}\right\}.$$

In addition, the electrostatic terms involving the SC atoms are modified as:

12a $${u}_{a}^{\mathrm{EL}}\left({r}_{ij};\lambda \right)=\frac{{q}_{i}{q}_{j}}{4\pi {\varepsilon }_{0}\sqrt{\beta \lambda +{r}_{ij}^{2}}},$$

12b $${u}_{b}^{\mathrm{EL}}\left({r}_{ij};\lambda \right)=\frac{{q}_{i}{q}_{j}}{4\pi {\varepsilon }_{0}\sqrt{\beta \left(1-\lambda \right)+{r}_{ij}^{2}}},$$

where ${q}_{i}$ and ${q}_{j}$ are the charges, and $\beta$ is a user-defined parameter. When $\beta =0$, ${u}_{a}^{\mathrm{EL}}$ and ${u}_{b}^{\mathrm{EL}}$ recover the standard Coulomb potential.

With the designs above, when $\lambda$ is 0 or 1, the $V\left(\overrightarrow{X};\lambda \right)$ in Eq. (10) becomes the potential energy of system $a$ or $b$, respectively. The systems with the intermediate $\lambda$ values thus establish an alchemical path between $a$ and $b$.

### Alchemical system for mutation of protein residues

Our task here is to evaluate the mutation of a single residue in the protein from type $a$ to type $b$, excluding proline (Pro) and any charge-reversing mutation (i.e., between a positively and a negatively charged types). The SC atoms (i.e., in ${\overrightarrow{X}}_{a}^{\mathrm{SC}}$ or ${\overrightarrow{X}}_{b}^{\mathrm{SC}}$) in our setup consist of the side chain atoms of the residue other than the C_β_, except when $a$ or $b$ is a Gly, in which case the SC includes the hydrogen atoms bonded to the C_α_ as well as the side chain of the non-Gly residue.

For charge-changing mutations, i.e., between a charged type and a neutral one, a co-alchemical ion is introduced to render the system electrically neutral for both end states. Specifically, the co-alchemical ion is a Na^+^ if the charged residue is negative, or a Cl^−^ if the residue is positively charged. In the end state corresponding to the neutral protein residue, the co-alchemical ion is assigned a zero charge, thus ensuring that the systems at both ends have identical total charge. The co-alchemical ion was initially placed in the bulk solution. In the simulations, we applied positional restraints (with a spring constant of 1 kcal/mol/Å^2^) on the co-alchemical ion as well as a C_α_ atom at the center of the protein, to ensure that the co-alchemical ion is always in the bulk region and has no close encounter with the protein.

### Preparation and equilibration of wildtype m396 systems

**Figure 2:**
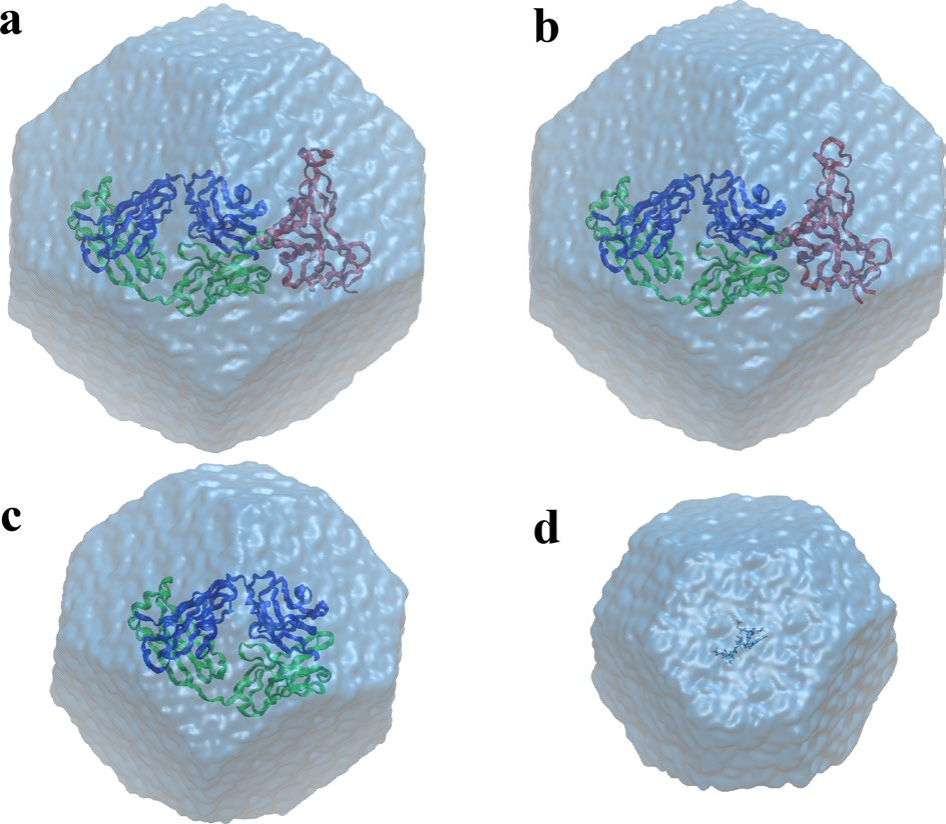
Four simulation systems in this study. (**a**) Complex of the m396 Fab and the SARS-CoV-1 RBD. (**b**) Complex of the m396 Fab and the SARS-CoV-2 RBD. (**c**) m396 Fab. (**d**) A 7-residue peptide representing the local structure of denatured antibody. Images were rendered by VMD^43^ (version 1.9.3).

In this study, FEP simulations were performed on four systems: (1) m396 bound to SARS-CoV-1 RBD; (2) m396 bound to SARS-CoV-2 RBD; (3) m396 alone; (4) 7-residue peptides taken from m396 or its variants, as models for denatured antibody. We describe the setup for each system (Fig. 2) below.

The protein complex of an m396 antigen-binding fragment (Fab) bound to the SARS-CoV-1 RBD was taken from the crystal structure^25^ 2DD8. The m396 Fab consists of a heavy chain and a light chain, folded into a variable domain and a constant domain. The RBD consists of residues 321–512 in the spike protein of SARS-CoV-1. The RBD and the Fab have 4 and 5 disulfide bonds, respectively. The glycan molecule attached to Asn330 of the RBD in the crystal structure^25^ was not included in our model. Standard protonation states at pH 7 were assigned to all the residues. In particular, all His residues were neutral, with the proton on the ε nitrogen (i.e., the “HIE” type in Amber^12^). The protein was solvated by 40,814 water molecules. In addition, 109 Na^+^ ions and 113 Cl^−^ ions were included to make the system electrically neutral and to establish a bulk NaCl solution of ~ 150 mM. The simulation system (Fig. 2a) consists of a total of 131,986 atoms. Periodic boundary conditions with a truncated octahedron unit cell were imposed in all the simulations.

The structure of the SARS-CoV-2 RBD was taken from the crystal structure^29^ 7BZ5, which has the best resolution among all the currently available structures. We superimposed this RBD against the SARS-CoV-1 RBD^25^ in 2DD8 to obtain a complex structure of the SARS-CoV-2 RBD and the m396 Fab. Specifically, the superimposition was based on the alignment of the C_α_ atoms in residues 336–456 and 491–516 of the SARS-CoV-2 RBD to the C_α_ atoms in residues 323–443 and 477–502 of the SARS-CoV-1 RBD. One missing residue in the crystal structure^29^, His519 of the RBD, was added back to the model based on another crystal structure^30^, 6M0J, of the complete SARS-CoV-2 RBD. Our simulation system (Fig. 2b) consists of residues 334–528 of the SARS-CoV-2 RBD, the m396 Fab, 41,248 water molecules, 110 Na^+^ ions and 115 Cl^−^ ions, with a total of 133,320 atoms. The structure (in pdb file) of this system after equilibration is provided in Supplementary Information.

The unbound antibody system was also built from the 2DD8 crystal structure^25^. The simulation system (Fig. 2c) consists of the m396 Fab, 22,777 water molecules, 60 Na^+^ ions and 62 Cl^−^ ions, with a total of 74,789 atoms. Crystal structures revealed that the conformations of the m396 variable domain are quite similar in the RBD-bound and the unbound states^25^. In addition, all the systems were subject to MD equilibration to allow the relaxation of the protein structure.

Each peptide system consists of the residue to be mutated as well as its 3 preceding and 3 succeeding residues in the antibody sequence, thus a total of 7 residues. The N- and C-termini of the peptide are capped by the [–C(=O)–CH_3_] and the [–NH–CH_3_] groups, respectively. Similar to other systems, the peptide was placed in a bulk solution of ~ 150 mM NaCl. Figure 2d shows one example of the peptide simulation system.

For each system, we first fixed all the protein atoms and performed 500 steps of conjugate-gradient minimization for the water and ions, followed by an MD equilibration of 2 ns. We then relaxed the protein and performed a minimization of 500 steps and an equilibration of 20 ns for the entire system. All the simulations in this stage were under a constant pressure of 1 atm. We then calculated the average of the system volume in the last 10 ns of the simulation trajectory and fixed the system volume at this average. All the subsequent FEP simulations were under constant volume. The length of the truncated octahedron unit cell (see Fig. 2) is ~ 120 Å for the RBD/Fab complex systems and 99.1 Å for the antibody system.

### FEP simulations for m396

In our protocols, the number of $\lambda$ windows is $N$=24 for charge-changing mutations and $N$=12 for others. The $\lambda$ values for the windows are evenly spaced between 0 and 1, including the two ends. For a mutation from the wildtype $a$ to a new type $b$, the input for the calculation is the equilibrated wildtype system described in “Preparation and equilibration of wildtype m396 systems”. For charge-changing mutations, we also introduce a co-alchemical ion (see “Alchemical system for mutation of protein residues”) in the simulation system as follows. Depending on whether the wildtype $a$ is charged or not, the co-alchemical ion is placed by taking an existing ion or by replacing a water molecule. For each ion or water molecule in the system, we calculate its shortest distance to any protein atom or their periodic images, and the ion/water with the longest distance is converted into the co-alchemical ion.

Our protocols consist of a forward calculation and a backward calculation. In the forward calculation, ${\lambda }_{1}=0$ corresponds to the residue type $a$ and ${\lambda }_{N}=1$ corresponds to the type $b$. We first carried out a “mutating” simulation, which starts at ${\lambda }_{1}$ and equilibrates the system at each ${\lambda }_{i}$ for 0.5 ns before changing $\lambda$ to the next value ${\lambda }_{i+1}$. In this way, the residue type was gradually transformed from type $a$ to type $b$ in the mutating simulation. Snapshots in this simulation were used to initiate the sampling simulations below.

In the production runs, all the $\lambda$ windows were sampled simultaneously, each by an individual simulation called a replica. Furthermore, Hamiltonian replica exchange^11^ was employed to allow neighboring windows to exchange their replicas. The criterion for swapping windows $i$ and $i+1$ is based on the change in the total energy due to the exchange: $\Delta E=\left[E\left({\overrightarrow{X}}_{i+1};{\lambda }_{i}\right)+E\left({\overrightarrow{X}}_{i};{\lambda }_{i+1}\right)\right]-\left[E\left({\overrightarrow{X}}_{i};{\lambda }_{i}\right)+E\left({\overrightarrow{X}}_{i+1};{\lambda }_{i+1}\right)\right]$, with the swapping probability given by^11^ $\mathrm{min}\left[\mathrm{exp}\left(-\Delta E/{k}_{B}T\right),1\right]$. The simulations were run for 5 ns per window unless noted otherwise, with the exchanges attempted every 0.4 ps. The last 4 ns of the simulation trajectories were used for free energy calculations.

As described earlier, the mutating simulation ended with the residue in type $b$. We further performed an equilibration of this mutant system for 10 ns. Starting from the equilibrated mutant, we carried out a backward calculation with the residue mutated from $b$ to $a$. Similar to the forward calculation above, the backward calculation involves a mutating simulation and subsequent sampling simulations with Hamiltonian replica exchange. The comparison of the forward and backward calculations serves as an important indicator for the convergence of the sampling, as will be elaborated in “Analysis and error estimation”.

### Common simulation protocols

We adopted the Amber ff14SB force field^31^ for the proteins and the TIP3P model^32^ for the water molecules. All simulations were run using a time step of 2 fs. All bond lengths involving hydrogen atoms were constrained using the SHAKE algorithm. Nonbond interactions were calculated with a cutoff distance of 8 Å. Full electrostatics was calculated using the Particle Mesh Ewald (PME) method^33^. A constant temperature of 300 K was maintained in all the simulations using the Langevin dynamics method with a collision frequency of 5 ps^−1^. We used the Amber software package^12^ to run the simulations on a computer cluster of IBM Power9 nodes equipped with NVIDIA TeslaV100 (Volta) GPUs. For technical reasons, we adopted an earlier version^12^ (Amber18) of the simulation engine (pmemd.cuda) in this study, which worked with better stability than Amber20 on our particular hardware and encountered fewer random crashes for the FEP simulations.

### Analysis and error estimation

At every 0.4 ps, when exchanges of the replicas were attempted, the Amber program^12^ output the potential energies $V\left({\overrightarrow{X}}_{i};{\lambda }_{i}\right)$ and $V\left({\overrightarrow{X}}_{i\pm 1};{\lambda }_{i}\right)$ for each window. These output data allowed us to calculate the ${\Delta E}_{ij}$ and ${\Delta E}_{ji}$ values in Eqs. (4)–(6). Among all the mentioned techniques (“Free energy difference between two systems” and “Sampling with intermediate windows”), we adopted the BAR method (Eq. (6)) to calculate the free energy differences between adjacent windows. We then added these incremental differences to obtain the free energy difference ${\Delta A}_{ab}\equiv {A}_{b}-{A}_{a}$ between the two end states.

However, it is not straightforward to directly derive the statistical errors in the BAR results. Therefore, we combined a few strategies to estimate the uncertainties. One strategy is to obtain the uncertainty from some of the alternative methods (see “Free energy difference between two systems” and “Sampling with intermediate windows”) that are easier for estimating the statistical errors therein. In addition, given that all those methods would give the same ideal ${\Delta A}_{ab}$ value if the sampling were infinite, the differences between their results could reflect to a lesser extent the deviation from the ideal ${\Delta A}_{ab}$ due to the finite sampling. Furthermore, a more important strategy is to estimate the uncertainty by comparing different simulations. The specifics of our error estimation are described below.

We first estimated the statistical errors in the EXP method (Eq. (4)), where the free energy difference ${\Delta A}_{ij}$ is obtained from the average of $\mathrm{exp}\left(-\frac{{\Delta E}_{ij}}{{k}_{B}T}\right)$ in window $i$. The variance of this average can be estimated by block averaging^34^ on the $\mathrm{exp}\left(-\frac{{\Delta E}_{ij}}{{k}_{B}T}\right)$ trajectory, and the variance in ${\Delta A}_{ij}$ can be further obtained. The sum of all the variances for ${\Delta A}_{ij}$ between adjacent windows was then taken as the variance of the end-to-end ${\Delta A}_{ab}$, and the square root of this variance was the standard error for the calculated ${\Delta A}_{ab}$. Furthermore, if the deviation between the ${\Delta A}_{ab}$ values calculated by the EXP (Eq. (4)) and the BAR (Eq. (6)) methods exceeds the standard error above, we would take this deviation as the new estimate for the standard error.

As the ${\Delta A}_{ij}$ may also be calculated by the EXP method based on the sampling of window $j$ (Eq. (5)), we followed the steps above to obtain another standard error. We then took the larger of the two standard errors as the statistical error estimated from the EXP method.

In addition, ${\Delta A}_{ab}$ can be calculated by the TI method (Eqs. (8) and (9)), which offers a straightforward uncertainty estimation. In particular, the variance of each ${f}_{i}$ may be estimated by block averaging^34^, and the statistical error of ${\Delta A}_{ab}$ can be further obtained. We took the larger of the error estimates from the EXP and the TI methods as the standard error for the FEP sampling.

For the forward and backward FEP calculations, following the procedure above, we obtained the ${\Delta A}_{ab}^{\mathrm{forward}}$ and ${\Delta A}_{ab}^{\mathrm{backward}}$ along with their variances $\mathrm{var}\left({\Delta A}_{ab}^{\mathrm{forward}}\right)$ and $\mathrm{var}\left({\Delta A}_{ab}^{\mathrm{backward}}\right)$, respectively. We always took the average $\left({\Delta A}_{ab}^{\mathrm{forward}}+{\Delta A}_{ab}^{\mathrm{backward}}\right)/2$ as our final result for ${\Delta A}_{ab}$. Accordingly, the standard error for the ${\Delta A}_{ab}$ would be $\sqrt{\mathrm{var}\left({\Delta A}_{ab}^{\mathrm{forward}}\right)+\mathrm{var}\left({\Delta A}_{ab}^{\mathrm{backward}}\right)}/2$. However, this estimated error will be overwritten by $\left|{\Delta A}_{ab}^{\mathrm{forward}}-{\Delta A}_{ab}^{\mathrm{backward}}\right|/2$ if the latter is larger. In such cases, the large deviation between the results from the forward and backward calculations will determine our statistical uncertainty.

### Protein thermal shift experiments

We performed experiments to measure the thermal stability for a group of selected m396 variants, which can be used to validate some of our FEP predictions. Humanized recombinant antibodies were purchased from Genscript. To determine melting temperatures, each antibody was subjected to protein thermal shift assay according to the manufacturer (ThermoFisher). Briefly, each antibody was diluted to 0.1 mg/ml in PTS buffer plus 1X PTS dye. Thermal shift was performed on an ABI 7500 Fast Real-Time PCR machine (ThermoFisher) according to the manufacturer recommendation of increasing the temperature from 25 to 99 °C at 1 °C per minute. Thermal profiles were analyzed using Protein Thermal Shift Software (ThermoFisher, version 1.4, https://www.thermofisher.com/order/catalog/product/4466038) using multi-peak analysis.

## Results

As described in “Methods”, we implemented automated protocols to evaluate antibody mutations using FEP and Hamiltonian replica exchange^11^ along with a systematic uncertainty estimation. In this section, we describe the application of the protocols in our effort of optimizing the m396 antibody. In our definition, a positive ${\Delta \Delta G}^{\mathrm{Binding}}$ (see Eq. (1)) means a weaker binding affinity compared to the wildtype, and a positive ${\Delta \Delta G}^{\mathrm{Stability}}$ (see Eq. (2)) means a poorer conformational stability for the antibody mutant.

### Particle collapse problem

**Figure 3:**
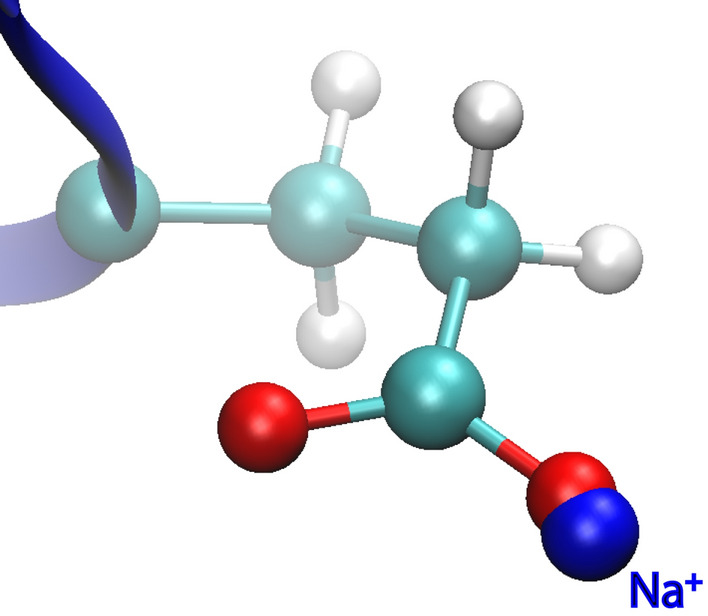
A snapshot from an FEP simulation with a Trp residue mutated to a Glu residue, in the window of $\lambda \hspace{0.17em}$= 0.65. The side chain of the Glu is shown, with an oxygen atom in very close contact to a Na^+^ ion from the bulk solution, thus exhibiting a particle collapse problem. Image was rendered by VMD^43^ (version 1.9.3).

Some of our preliminary calculations involving the Asp or Glu residues exhibited abnormally large statistical errors, thus indicating severe sampling problems. Visual inspection of the corresponding simulation trajectories revealed a “particle collapse problem”^4^, with an SC oxygen atom in the carboxylic group of Asp or Glu making abnormally close contact with a Na^+^ ion from the bulk solution, as illustrated in Fig. 3. This behavior could be explained by Eqs. (11) and (12) for the interaction between the two overlapping atoms. In these equations, the default Amber parameters are $\alpha$ = 0.5, $\beta$ = 12 Å^2^, and the force field parameters for the pair of oxygen and Na^+^ atoms are ${q}_{i}$ = − 0.8188 e, ${q}_{j}$ = 1 e, and $\varepsilon$ = 0.1355074 kcal/mol. With these parameters, a complete overlap of the two atoms (i.e., ${r}_{ij}=0$) in the system with $\lambda$ = 0.5 corresponds to a VDW energy (Eq. (11)) of 6.5 kcal/mol and an electrostatic energy (Eq. (12)) of − 111.2 kcal/mol, or a net interaction energy (Eq. (10)) of − 52.3 kcal/mol. In the default setting, therefore, a collapse of the two atoms into each other is energetically favorable^35^.

In principle, the unphysical behaviors in the intermediate $\lambda$ windows should not affect the correctness of the calculated free energy difference between the two ends. In this case, however, ion association and dissociation have very slow kinetics, and the presence or absence of the ion has a significant effect on the energies. Consequently, it is difficult to fully equilibrate some of the intermediate $\lambda$ windows, thus resulting in very large statistical errors.

In the SC potentials (Eqs. (11) and (12)), the parameter $\alpha$ controls the softness of the particle core, and $\beta$ determines the limit of the electrostatic energy at short distances. To solve the particle collapse problem, therefore, one may decrease $\alpha$ to make the VDW energy more repulsive and thus make the atoms “harder”, or increase $\beta$ to reduce the attractiveness of the electrostatic energy. In this study, we reduced $\alpha$ from the default value of 0.5 to 0.3 for all the FEP simulations. The new $\alpha$ value is also within the examined range in a previous systematic evaluation^35^. In addition, mutations between Asp and Glu appeared to be more vulnerable to the problem, and we thus further increased the $\beta$ parameter from the default of 12 Å^2^ to 20 Å^2^ and increased the number of windows from 12 to 24 for such cases. After the adjustment of the SC parameters, the particle collapse problem no longer appeared to be significant in our simulations.

### Evaluating single mutations

We applied our automated FEP protocols to exhaustively evaluate single mutations for 27 residues located on the m396 binding interface to the RBD. Each of the wildtype residues was mutated to all the other types except Pro and charge-reversing ones. In total, this task involved 480 single mutations from the wildtype m396. For each mutation, we calculated the $\Delta G$ values for the complex systems of m396 bound to SARS-CoV-1 and SARS-CoV-2 RBDs, the isolated m396 system, and the 7-residue peptide system (Fig. 2).

**Figure 4:**
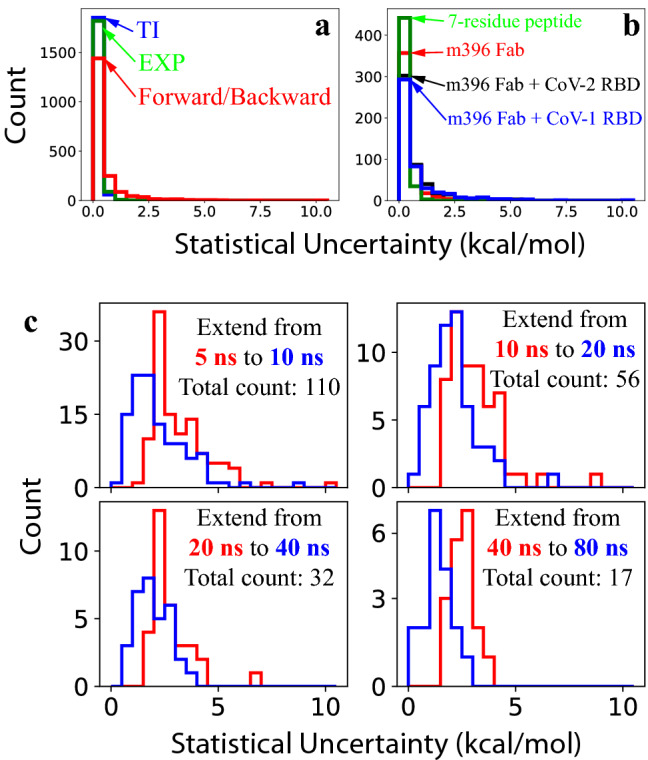
Distributions of the statistical uncertainties in the $\Delta G$ calculations. (**a**) Histograms for the statistical errors estimated by three different methods, i.e., EXP, TI, and difference between the forward and backward simulation results, as described in “Analysis and error estimation”. (**b**) Histograms of the statistical errors in the original calculations, with 5 ns per window, for the four systems. (**c**) Comparisons of the uncertainty histograms after extending some of the sampling simulations from 5 to 10 ns, from 10 to 20 ns, from 20 to 40 ns, and from 40 to 80 ns.

We also performed uncertainty estimation for each $\Delta G$ calculation. As explained in “Analysis and error estimation”, our statistical error was essentially taken as the largest among three estimates based on the EXP method, the TI method, and the comparison of forward and backward simulations, respectively. Figure 4a shows the distributions of the errors estimated by the three individual methods for all the $\Delta G$ calculations. Whereas the EXP and TI methods provided similar statistical errors overall, the difference between results from the forward and backward FEP simulations revealed substantially larger errors in many cases. Indeed, most of the large estimated uncertainties here were due to the deviation between the forward and backward results, thus confirming that our method of comparing simulations starting from distinct initial coordinates would offer a more stringent error estimation than methods based on single simulations do^36^.

Figure 4b displays the distributions of the estimated standard errors for the $\Delta G$ calculations in the four systems. Although the sampling simulations (5 ns per window) were relatively short, most of our calculations nonetheless had satisfactory statistical errors. Overall, the statistical errors here appear to be negatively correlated to the structural complexity of the system, with the simplest 7-residue peptide system having the smallest uncertainty and the antigen–antibody complex systems having the largest uncertainties.

For the ${\Delta \Delta G}^{\mathrm{Binding}}$ and ${\Delta \Delta G}^{\mathrm{Stability}}$ calculations in this study, we aimed to achieve a statistical accuracy of 2 kcal/mol or better. For the cases in which the statistical error exceeded 2 kcal/mol, therefore, we extended the sampling simulations from 5 to 10 ns per window. Subsequently, for the cases with the errors still above 2 kcal/mol in the 10-ns sampling, we further extended the simulations to 20 ns per window. Similarly, we performed extension runs for the subset of simulations with large errors to 40 ns and then to 80 ns. As shown in Fig. 4c, each extension clearly reduced the overall statistical errors in the $\Delta G$ calculation and reduced the number of cases with unacceptable uncertainty. After all the extensions (up to 80 ns per window), the vast majority of our calculated ${\Delta \Delta G}^{\mathrm{Binding}}$ and ${\Delta \Delta G}^{\mathrm{Stability}}$ values had statistical errors below 2 kcal/mol. Calculation results for all the single mutations are provided in Supplementary Information.

**Figure 5:**
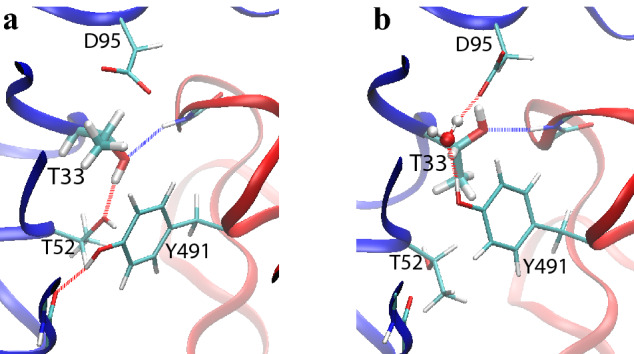
Snapshots of two alternative side chain conformations for the residues around Thr33 in the binding interface between the m396 heavy chain (blue) and the SARS-CoV-1 RBD (red). H-bonds between the residues are shown. In panel (**b**), a water molecule is also involved in the H-bond network. A proper equilibration between these alternative conformations would require long simulation times, thus resulting in slow convergence of the FEP calculations. Images were rendered by VMD^43^ (version 1.9.3).

Several $\Delta G$ calculations still had relatively large statistical errors after the extensive sampling (80 ns per window), mainly due to the presence of alternative conformations of the protein residues. Figure 5 shows such an example taken from the FEP simulations for the complex of m396 and SARS-CoV-1 RBD, with the wildtype Thr33 in the m396 heavy chain mutated to an Asp. In the simulations, Thr33 and nearby residues exhibited multiple conformations, two of which are shown in the figure. The conformations differ in the sidechain rotamer states of Thr33 and Thr51 as well as the local H-bond network. Furthermore, spontaneous transitions between these conformations were rare in the simulations, and proper equilibration of the conformations was thus difficult and would require very long simulation times. Consequently, the statistical uncertainty here was relatively high, as manifested by the deviation between the results from the forward and backward calculations. Such conformational variability is a common cause of slow convergence and large uncertainty in the FEP simulations. One potential solution to the problem would be to run a separate FEP calculation for each of the conformational states (e.g., each rotamer). However, to properly combine the results and obtain the overall $\Delta G$ between the two end states, we also need to know the equilibrium probabilities of the alternative conformations in at least one end state. If spontaneous transitions between these conformations are too rare, other enhanced sampling techniques^36^ could be employed to calculate such probabilities.

### Evaluating multi-point mutants

Antibody design typically involves mutations of multiple residues from the wildtype template, and the capability of FEP to evaluate multi-point mutants is thus desirable. Given that our protocols take one mutation at a time, we adopted a multi-step approach to handle the multi-point mutants. For example, the free energy shift for a double-mutant $\left(a,b\right)$ relative to the wildtype (denoted as 0 below) is equal to the sum of two stepwise shifts:

13 $${\Delta \Delta G}_{0\to a,b}={\Delta \Delta G}_{0\to a}+{\Delta \Delta G}_{a\to a,b}={\Delta \Delta G}_{0\to b}+{\Delta \Delta G}_{b\to a,b},$$

where the first step is between the wildtype and an intermediate single-point mutant, and the second step is between the single-point mutant and the target double-point mutant. With this approach, we evaluated a given set of 221 mutants of m396, each consisting of mutations on two to twelve residues. Here we do not discuss how the mutants were proposed, and instead only focus on our FEP evaluation of these provided antibodies for their relative conformational stability and binding affinity to the SARS-CoV-2 RBD.

In principle, each $n$-point mutant should take $n$ steps to evaluate. However, because different mutants may share common single mutations, a proper scheduling could maximize the number of shared intermediates and thereby reduce the total number of the steps to evaluate all the given mutants. In this study, we adopted a greedy heuristic below to minimize the total computational cost. First, similar to the Hamming distance, we defined the distance between two mutants as the number of residues that are different between the two. For example, the distance between the wildtype and an $n$-point mutant is $n$. We then created an “evaluation set” of mutants and defined the distance from any mutant $x$ to the evaluation set as the shortest distance from $x$ to any member of the set. The sum of the distances from the 221 target mutants to the evaluation set was denoted as $L$. Initially, the evaluation set contained the wildtype only, giving rise to $L$=1,410 for our case here. Next, given that we already evaluated 480 single mutations (see “Evaluating single mutations”), we added the corresponding 480 single-point mutants to the evaluation set, which reduced $L$ to 1,189. Subsequently, we added new mutants to the evaluation set one at a time. At each step, the new mutant must be derived from an existing member in the evaluation set by mutating one more residue. Among all the eligible new mutants that can be added for the current step, we chose the one that minimizes the $L$ for the resulting new evaluation set. Such steps were repeated until $L$ was reduced to zero, when all the targets were in the evaluation set. With this strategy, it took 554 steps to reduce $L$ from 1,189 to 0, with each step evaluated by an individual FEP calculation. The $\Delta \Delta G$ for any target mutant with respect to the wildtype was then obtained by summing up the $\Delta \Delta G$ values from the individual steps.

Alternatively, by assuming additivity^37^, the $\Delta \Delta G$ for a given multi-point mutant can be approximated by adding the $\Delta \Delta G$ values for the individual single mutations from the wildtype. For example, the $\Delta \Delta G$ for the double-mutant in Eq. (13) can be approximated as ${\Delta \Delta G}_{0\to a,b}\approx {\Delta \Delta G}_{0\to a}+{\Delta \Delta G}_{0\to b}$. With the $\Delta \Delta G$ values available for all the single mutations, this approximation allows a direct estimate of the $\Delta \Delta G$ for any given multi-point mutant without running additional simulations.

**Figure 6:**
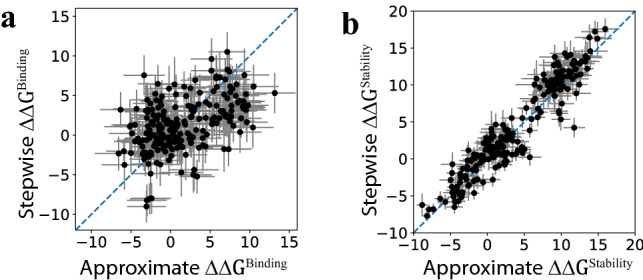
Comparisons of the $\Delta \Delta G$ values for 221 multi-point mutants obtained from two methods, as described in the text. The approximate $\Delta \Delta G$ for the mutant is the sum of the $\Delta \Delta G$ values for the corresponding single mutations with respect to the wildtype. The stepwise $\Delta \Delta G$ for the mutant is calculated by adding the incremental $\Delta \Delta G$ values when the wildtype is transformed to the mutant one residue at a time. The $\Delta \Delta G$ is shown for both the binding to the SARS-CoV-2 RBD (**a**) and the stability of the mutant (**b**). All the units are in kcal/mol.

As shown in Fig. 6, we examined the additivity by comparing the approximate $\Delta \Delta G$ values above to the more rigorous $\Delta \Delta G$ obtained from the stepwise FEP calculations described earlier. Overall, for ${\Delta \Delta G}^{\mathrm{Stability}}$ (Fig. 6b), the two methods agree reasonably well, thus suggesting that the ${\Delta \Delta G}^{\mathrm{Stability}}$ values for the single mutations are roughly additive and can be used to approximately evaluate the conformational stability of a multi-point mutant. In the meantime, we caution that the stability calculations here were based on highly simplified models for the denatured state, and experimental measurements are still required to confirm our finding for the additivity of ${\Delta \Delta G}^{\mathrm{Stability}}$. In contrast, the agreement is much poorer for the ${\Delta \Delta G}^{\mathrm{Binding}}$ (Fig. 6a), thus indicating that these residues at the binding interface may not act completely independently and that the ${\Delta \Delta G}^{\mathrm{Binding}}$ values for the single mutations may not be quantitatively predictive for their collective effects on the binding affinity.

**Figure 7:**
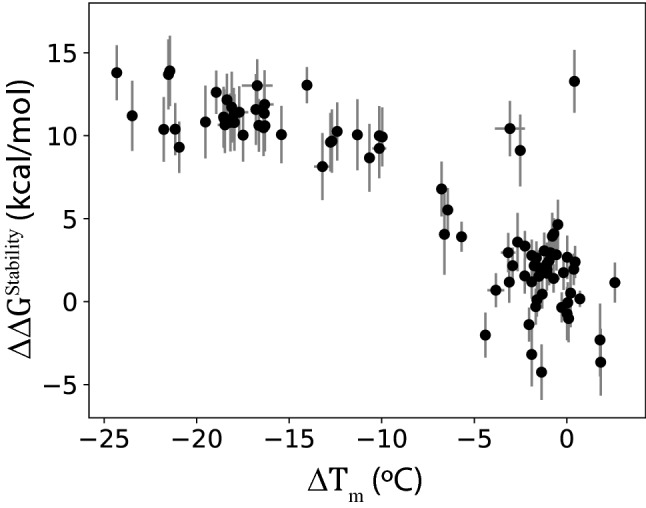
The predicted ${\Delta \Delta G}^{\mathrm{Stability}}$ for the m396 multi-point mutants from the FEP simulations vs. the shift of their experimentally measured melting temperature relative to the wildtype m396. Some of the horizontal error bars are smaller than the size of the data marker and are thus invisible.

We also experimentally measured the melting temperature (${T}_{m}$) for some of the m396 mutants above (see “Protein thermal shift experiments”). Most of the mutants exhibited two peaks in the thermal shift curve^38^, in which cases we took the first peak as the ${T}_{m}$. We performed four measurements for each mutant and took the mean and the standard error as the reported ${T}_{m}$ and its uncertainty, respectively. All the computational results and the ${T}_{m}$ data for the m396 multi-point mutants are provided in Supplementary Information. Figure 7 shows the change ($\Delta {T}_{m}$, relative to the wildtype) in the experimental melting temperature vs. the FEP-predicted ${\Delta \Delta G}^{\mathrm{Stability}}$ for each tested variant. Although simplified theories^24,39^ predicted a linear relation between ${\Delta \Delta G}^{\mathrm{Stability}}$ and $\Delta {T}_{m}$ for small changes (i.e., when the two are close to zero), their relation over large ranges has no known quantitative form and would be system specific. Therefore, we did not attempt to fit the data points in Fig. 7 into a mathematical (such as linear) function. Rather, some qualitative classification would be more practical for this dataset. Indeed, most of the mutants with small ${\Delta \Delta G}^{\mathrm{Stability}}$ also have small $\Delta {T}_{m}$, thus consistently indicating similar stabilities relative to the wildtype. Moreover, with a few exceptions, the mutants with large positive ${\Delta \Delta G}^{\mathrm{Stability}}$ tend to have much lower ${T}_{m}$ values, thus indicating significantly reduced stability.

Given the additivity described earlier, the ${\Delta \Delta G}^{\mathrm{Stability}}$ of a multi-point mutant can be approximated by the sum of contributions from individual single mutations. This analysis allowed us to identify the mutation of Trp47 in the heavy chain to Asp as the major contributor for the large predicted ${\Delta \Delta G}^{\mathrm{Stability}}$. Indeed, all the antibodies with this W47D mutation were predicted a highly positive ${\Delta \Delta G}^{\mathrm{Stability}}$. However, although most of these mutants do have significantly reduced ${T}_{m}$ values as expected, there are three clear outliers (at the upper-right corner of Fig. 7) with their ${T}_{m}$ not very different from that of the wildtype. One possible reason for the discrepancy might be that those mutants could establish a somewhat differently folded conformation which is more probable but failed to be sampled by our MD simulations. In addition, the simplification in our models of denatured conformation could always be a source of inaccuracy. Further experiments on relevant variants are thus required to elucidate the underlying cause. Nonetheless, our calculations correctly identified the potentially destabilizing mutation of W47D, thus providing helpful information for the antibody design.

## Discussion

In this article, we report our implementation of automated FEP calculations to facilitate antibody design. Our protocols enable systematic large-scale predictions, such as scanning the residues in the binding interface and exhaustively evaluating all possible single mutations for these residues. A critical component in our protocols is a faithful estimation of the statistical uncertainty in the results. The uncertainty would determine the level of confidence when the individual results are considered in the decision making. Furthermore, abnormally high uncertainty could indicate pathological cases, such as the particle collapse problem identified in this study. Such alarms are especially important in the automated large-scale calculations, where manual inspections on every individual case become unfeasible. In addition, a faithful uncertainty estimation could identify the simulations that need to be improved. In this study, e.g., whereas most FEP simulations were relatively short, we extended only those simulations with large statistical errors to much longer sampling times.

Due to the stochastic nature of the sampling, no method guarantees correct uncertainty estimation for all cases^36^. For example, although most of the extended samplings reported in this study reduced the estimated uncertainty, the error estimates for a small fraction of the simulations became even larger after the extension. Therefore, it is entirely likely that among the simulations that were not extended, the uncertainty of some may have been underestimated and would have been revealed in extension runs. As another example, in our evaluation of the multi-point mutants (“Evaluating multi-point mutants”), each mutant is connected to the wildtype by a single path of intermediates, whereas establishing multiple paths could improve the uncertainty estimation by exploiting the thermodynamic consistency^2^. In general, longer and more simulations should always make the results more reliable. In practical applications, however, the increased computational cost associated with more samplings has to be considered as well. In this study, by allocating the computational resource to the cases with the most needs, most of our calculations appeared to achieve acceptable statistical uncertainties at a modest aggregated simulation time. Our approach thus strikes a good balance between the statistical accuracy and the computational cost.

An important strategy in our uncertainty estimation is to compare the results from two groups of FEP simulations starting from different initial coordinates, namely, a forward calculation from residue $a$ to $b$ and a backward calculation from $b$ to $a$. In contrast to estimating errors from single simulations alone, this approach could often capture statistical uncertainties (see Fig. 4a) caused by slow equilibrations^36^, such as the case shown in Fig. 5 with multiple alternative conformations of the protein residues.

In the context of rapid large-scale screening here, we considered it acceptable if the statistical uncertainty is below 2 kcal/mol. In the meantime, we note that with sufficient sampling, FEP can often achieve a higher accuracy, e.g., 1 kcal/mol or even better. If an improved statistical accuracy is needed, however, we may always further extend the FEP sampling to reduce the uncertainty, as similarly done in this study. Furthermore, as experimental binding affinities are not available yet at this stage, we do not have a direct validation for our predicted ${\Delta \Delta G}^{\mathrm{Binding}}$ values and do not know their actual errors.

In addition to the relative binding affinity, our protocols also predict the relative conformational stability for the antibody mutant. Protein stability describes the equilibrium between its native (folded) and denatured (unfolded) conformations. Given that the denatured structure is not known, one needs to resort to highly simplified models to approximate the local environment of the mutated residue in the denatured conformation. In many FEP works, e.g., the denatured state was represented by just a single amino acid (i.e., the residue under the mutation) in bulk solution. Recent studies^18^ demonstrated that the accuracy of the predicted stability can be improved if a few flanking residues are also included in the model for the denatured state, as adopted in this study. Whereas our peptide system remains a crude model for the denatured protein, our predicted free energy shift for the measured m396 mutants appears to be consistent with the experimental melting temperature for the majority of the test cases. Furthermore, predictions of both the affinity and the stability by our FEP protocols would allow one to jointly consider these factors in the early stage of antibody design, thus helping develop antibodies with good manufacturability properties that will become important for the clinical applications at a later stage.

As FEP has been widely adopted in many applications, there are multiple variations in its implementation. In the following, we will briefly discuss the tradeoffs for some of the alternative implementations and suggest potential improvement for future applications.

First, as the case for all MD-based methods, the accuracy of FEP heavily replies on the faithfulness of the simulation system, especially the quality of the protein structures. In addition, the protonation states of the protein residues may also have a significant impact on the simulation. In this study, we adopted the simplest approach of assigning standard protonation state for all the residues. More careful treatment of the protonation states, such as by PROPKA^40^ or other programs, should in principle improve the accuracy of our FEP calculations.

As mentioned earlier, longer and more simulations should always improve the statistical uncertainty but will increase the computational cost. Therefore, alternative implementations should be compared based on the same computational cost determined by the total simulation length. For example, one could repeat the same sampling simulation multiple times with different initial velocities^41^, or alternatively extend the sampling multiple times longer. Here we would argue that a longer simulation is often preferred over multiple short runs. The essence of equilibration is to “forget” the initial microstate during the simulation, such that the statistics become completely independent of the particular starting coordinates^36^. In this aspect, a long simulation should more likely forget the initial condition than multiple short simulations do, especially when involving a relaxation with nontrivial first passage times to some more probable structures. As demonstrated in this study, the discrepancy between our forward and backward simulations became increasingly smaller as the simulations were extended longer, and it is doubtful that the same could be achieved by repeatedly running those simulations but with a fraction of the sampling time for each run. On the other hand, when equilibration is fast and not of a concern, running multiple shorter simulations has the advantage of parallel execution and thus possibly a faster turnaround.

A related issue concerns the number and the placement of the $\lambda$ windows in FEP. In this study, we adopted a simple recipe^5,6^ of 12 or 24 windows depending on whether the mutation is charge-changing or not. However, we found that with the same number of windows, calculations involving bulky residues (such as Trp) normally have substantially higher statistical uncertainties than others, thus suggesting that it would be beneficial if the number of $\lambda$ windows is determined according to the residue type, with more windows allocated to larger residues. Furthermore, with Hamiltonian replica exchange implemented, the exchange rate between adjacent windows could conveniently indicate whether the windows have sufficient overlap. Indeed, the accuracy of the calculated $\Delta A$ (see “Free energy difference between two systems” and “Sampling with intermediate windows”) between two neighboring windows appears to be correlated to their exchange rate. Moreover, we observed that the exchange rates for the windows near the ends (i.e., when $\lambda$ is near 0 or 1) tend to be substantially lower than those in the midrange, especially after our adjustment of the $\alpha$ and $\beta$ parameters (“Particle collapse problem”). Therefore, instead of uniformly placing the $\lambda$ windows as in this study, the sampling efficiency may be improved by allocating denser windows near the ends and sparser windows in the midrange.

Our FEP implementation in this study adopted the one-step transformation where the electrostatic and VDW potentials vary simultaneously (Eqs. (11) and (12)) with $\lambda$. In contrast, multi-step transformation is an alternative scheme in which the SC atoms are decharged prior to the transformation of their VDW interactions^35,41^. Although technically the one-step transformation is slightly simpler, the multi-step transformation has the advantage of eliminating the particle collapse problem encountered here.

The double-system/single-box setup^42^ is an alternative simulation setup for FEP, in which two entities (such as one antibody and one peptide) are placed far apart from each other in the same simulation system. Then in the FEP simulations, the alchemical transformation from $a$ to $b$ in one entity occurs simultaneously with the transformation from $b$ to $a$ in the other entity. Despite a much larger system size and higher atom count for the simulations, this setup could obtain the $\Delta \Delta G$ in a single FEP calculation, whereas in the traditional setup the $\Delta \Delta G$ is the difference between two $\Delta G$ s (Eqs. (1) and (2)), each from a separate FEP calculation. For charge-changing mutations, the double-system/single-box setup has a unique advantage of simultaneously satisfying the charge neutrality in both ends, thus making the co-alchemical ion unnecessary. In this study, we still adopted the traditional setup, partly due to its technical simplicity. In addition, as mutation on the isolated antibody is required for determining multiple $\Delta \Delta G$ s here, it is more convenient to obtain the ${\Delta G}^{\mathrm{Antibody}}$ (Eqs. (1) and (2)) in just one FEP calculation.

In this article, we mainly focus on the FEP technique, using m396 as a case study. As mentioned in “Introduction”, our FEP implementation is a component in a much broader multidisciplinary effort of antibody design. Our project involves several other antibodies, a diverse set of computational approaches for proposing and evaluating antibody mutants, and extensive experiments. The other components of the project will be presented in forthcoming reports.

In summary, given the capability of FEP to rigorously predict relative free energies, our work here could help transform this powerful technique into a reliable and efficient tool that can be routinely applied to aid antibody design and to elucidate protein–protein interactions.

## Supplementary Information

Supplementary Information 1.

Supplementary Information 2.

Supplementary Information 3.

Supplementary Information 4.

## Data Availability

Computational and experimental data are provided in Supplementary Information. Relevant code (scripts) may be requested from the corresponding author.
