# Supplementary material for: Large-scale application of free energy perturbation calculations for antibody design
Source: Sci Rep. 2022 Jul 21;12:12489. doi: 10.1038/s41598-022-14443-z (PMC9302960; doi:10.1038/s41598-022-14443-z)
Supplement: Supplementary file 3 — Supplementary Information 3. [file 41598_2022_14443_MOESM3_ESM.pdf]

Large-scale application of free energy perturbation calculations for antibody design

Fangqiang Zhu[1], Feliza A. Bourguet[1], William F. D. Bennett[1], Edmond Y. Lau[1], Kathryn T. Arrildt[1], Brent W. Segelke[1], Adam T. Zemla[2], Thomas A. Desautels[3], Daniel M. Faissol[3]

1. Biosciences and Biotechnology Division, Physical and Life Sciences Directorate, Lawrence Livermore National Laboratory, USA
2. Global Security Computing Division, Computing Directorate, Lawrence Livermore National Laboratory, USA
3. Computational Engineering Division, Engineering Directorate, Lawrence Livermore National Laboratory, USA

The Supplementary Information includes the following files:

- m396+Cov2.pdb: Equilibrated simulation system for the complex of m396 Fab and the Sars-Cov-2 RBD
- SingleMutation.csv: All computational results regarding the single mutations on m396
- Multipoint.csv: All computational results for 221 multi-point mutants of m396, some of which also have experimental melting temperatures
